# Emissions of Polycyclic Aromatic Hydrocarbons (PAHs) from the Use of Scented Candles Under Different Environmental Conditions

**DOI:** 10.3390/toxics14070565

**Published:** 2026-06-27

**Authors:** Chun-Yu Chen, Chiao-Ling Shih, Yu-Chieh Kuo, Perng-Jy Tsai

**Affiliations:** 1 Department of Environmental and Occupational Health, National Cheng Kung University, 138 Sheng-Li Rd., North Dist., Tainan 70403, Taiwan11502035@gs.ncku.edu.tw (C.-L.S.); 2 Department of Occupational Safety and Health, Chung Hwa University of Medical Technology, 89 Wenhua 1st St., Rende Dist., Tainan 71703, Taiwan

**Keywords:** scented candles, polycyclic aromatic hydrocarbons, emission characteristics, health risk assessment

## Abstract

This study investigated the effects of environmental conditions on polycyclic aromatic hydrocarbon (PAH) emissions from scented candles during combustion. An exposure chamber was established and validated to ensure stability and uniformity before the experiments were conducted. One of the most widely used scented candles (paraffin wax + 6% lavender essential oil) was selected in the present study. Testing environmental conditions included three relative humidity (RH) levels (60%, 75%, and 90%) and three air exchange rates (ACHs) (0.5, 1.0, and 2.0 h^−1^). For each tested environmental condition, three replicate measurements were conducted. Gas-phase and particle-bound polycyclic aromatic hydrocarbons (PAHs) were collected using filter cassettes and XAD-2 sorbent tubes, respectively. Results showed that, under identical RH conditions, both C_total-PAHs_ and EF_total-PAHs_ followed the trend of 0.5 ACH > 2.0 ACH > 1.0 ACH, whereas total-Bap_eq_ and EF_Total-BaPeq_ values decreased with increasing ACH. While under identical ACH conditions, emissions showed a nonlinear response to RH, following the trend: 75% > 90% > 60%. Most detected PAHs were present in the gaseous phase and were dominated by low-molecular-weight compounds containing two to three aromatic rings. The estimated highest incremental lifetime lung cancer risk reached 1.20 × 10^−6^ for aromatherapy workers, assuming an exposure duration of 8 h day^−1^ over 40 years. These findings highlight the potential health risks associated with the use of scented candles and emphasize the importance of adequate ventilation to reduce long-term indoor exposure.

## 1. Introduction

Scented candles are widely used in indoor environments for decorative, relaxation and aromatherapy purposes. The increasing popularity of aromatherapy and demand for indoor fragrances have increased the use of scented candles in recent years [1]. The global scented candle market was estimated at approximately 2.24 billion US dollars in 2024 and is expected to reach 3.47 billion US dollars by 2030 [2]. Scented candles are primarily composed of solid wax mixtures and fragrance oils. Common wax materials include paraffin wax, beeswax, soy wax, and palm wax [3,4,5]. Among these materials, paraffin wax is the most widely used wax base due to its relatively low cost and widespread availability. Paraffin wax is derived from petroleum distillation and primarily consists of long-chain hydrocarbons. Fragrance additives typically contain volatile organic compounds (VOCs) and aromatic substances [6,7], including both steam-distilled plant oils (e.g., lavender, eucalyptus, peppermint, tea tree, and lemon) and blended fragrance oils containing synthetic aroma chemicals, natural extracts, and essential oils.

Although scented candles are commonly considered safe household products, their combustion has been recognized as a potential source of indoor air pollutants [8,9,10]. During burning, incomplete combustion of candle wax and fragrance additives can generate a complex mixture of indoor pollutants, including fine and ultrafine particulate matter, soot, carbon monoxide, polycyclic aromatic hydrocarbons (PAHs), and a range of volatile organic compounds and carbonyls such as benzene, toluene, and formaldehyde, several of which are recognized carcinogens or irritants [11,12,13]. Thermal cracking and incomplete oxidation of hydrocarbons in paraffin wax can produce aromatic intermediates, which may subsequently form PAHs through pyrolysis reactions [14]. Under conditions of insufficient ventilation, these pollutants may accumulate indoors. This results in elevated exposure concentrations and potential health risks. This concern is particularly important for environments such as aromatherapy treatment rooms, where scented candles may be continuously used, and ventilation is intentionally limited to preserve fragrance.

Previous studies have demonstrated that several 4–7 ring PAHs are carcinogenic, precarcinogenic, or cocarcinogenic compounds [15]. In particular, benzo[*a*]pyrene (BaP) has been classified as a human carcinogen by the International Agency for Research on Cancer [16]. Long-term exposure to PAHs may therefore increase carcinogenic risk in indoor environments. Several studies have investigated PAH emissions from candle combustion under controlled indoor conditions. Lau et. al. [17] evaluated paraffin, stearic, and beeswax candles in an exposure chamber under fixed oxygen concentrations and non-turbulent combustion conditions, reporting PAH and BaP emission factors ranging from 4.75 to 156 ng g^−1^ and from 0.01 to 0.13 ng g^−1^, respectively. Fine et al. [18] examined paraffin and beeswax candles at a fixed ventilation rate of 100 L min^−1^ and found that PAH emission rates during normal burning, sooting combustion, and smoldering conditions were all below 0.1 mg g^−1^. Wallace [19] further investigated PAH emissions from 11 commonly used scented candles using real-time monitoring methods and reported that citronella-scented candles produced the highest PAH emissions.

Despite all these findings, previous studies generally assumed fixed environmental conditions and did not systematically evaluate the influence of ventilation and humidity on PAH formation and emissions. Environmental factors such as air exchange rate (ACH) and relative humidity (RH) may significantly influence combustion chemistry, pollutant formation, and gas-particle partitioning of PAHs [20,21,22]. For example, studies on incense combustion have shown that increasing relative humidity can reduce PAH emissions [23], yet comparable investigations for scented candles remain limited. Therefore, the combined effects of air exchange rate and relative humidity on PAH emission from scented candle combustion remain inadequately investigated.

Accordingly, this study first established and validated an exposure chamber to ensure stability and uniformity of experimental conditions. The chamber was then used to investigate scented candle combustion under different ACH and RH, with emphasis on PAH composition, gas-particle partitioning, emission rate, and emission factor. Finally, the results were applied to estimate the incremental lifetime lung cancer risk associated with PAH exposure among aromatherapy workers under different environmental conditions, thereby providing a basis for evaluating potential health risks associated with scented candle use.

## 2. Materials and Methods

### 2.1. Establishing the Exposure Chamber

**Figure 1:**
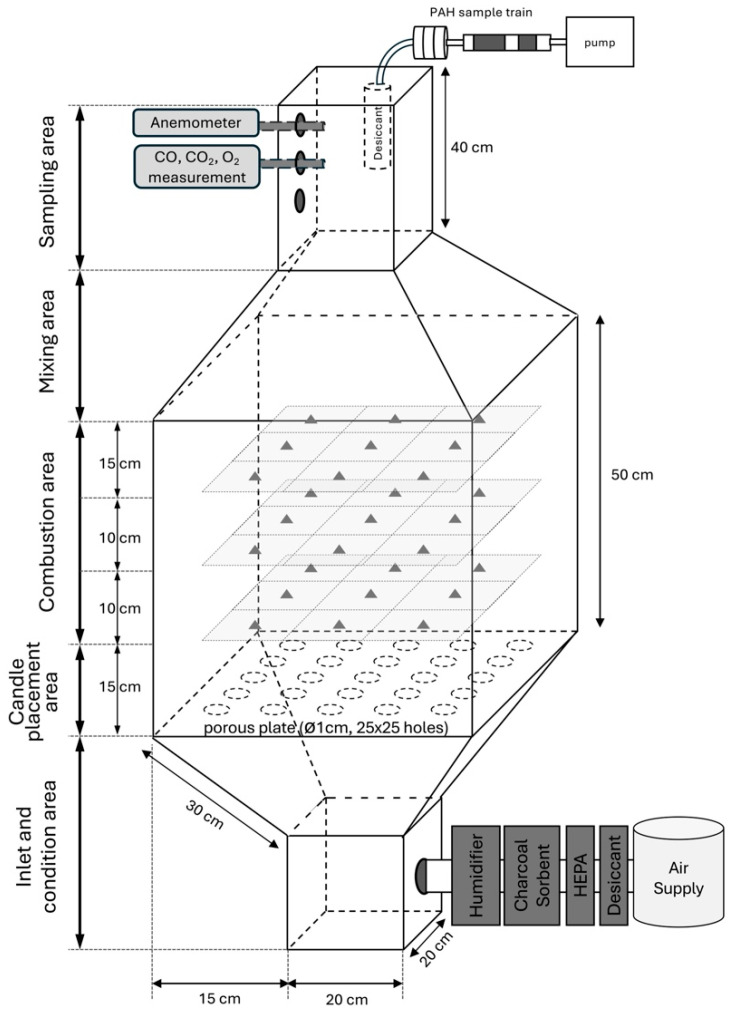
The established exposure chamber.

A custom acrylic exposure chamber was constructed to provide stable and reproducible environmental conditions for scented candle combustion tests. The chamber consisted of five functional zones: an inlet section, a candle placement section, a combustion testing section, a mixing section, and a sampling section (Figure 1). The inlet section was connected to an air supply system for steady conditioned air. A porous plate was installed beneath the candle placement section to improve flow uniformity before air entered the combustion section. The combustion section provided a controlled area for the stable combustion of scented candles. The mixing section allows emitted pollutants to mix thoroughly before sampling. The sampling section, in which sampling ports were installed for the measurement of PAHs and air composition.

The air supply system of the exposure chamber was connected to an external compressed-air system to provide the required ventilation rate during the experiments. A high-efficiency particulate air (HEPA) filter, activated carbon adsorption unit, and dehumidification unit were installed upstream of the air inlet to remove particles, gaseous contaminants, and moisture from the incoming air. In addition, a humidification unit was installed to regulate the relative humidity of the supplied air.

### 2.2. Stability and Uniformity Testing

Before candle combustion experiments, the chamber was evaluated for temporal stability and spatial uniformity of RH and ACH. Temporal stability was measured at the center of the combustion section. Spatial uniformity was evaluated at three sampling layers, with 9 locations per layer, giving 27 measurement points. Two condition sets were tested: a fixed ACH of 0.5 h^−1^ and target RH values of 60%, 75%, and 90%, and a fixed RH of 75% and target ACH values of 0.5, 1.0, and 2.0 h^−1^.

RH and ACH were recorded repeatedly over five hours for the stability test. Before each uniformity test, the chamber was allowed to equilibrate. RH was measured using a humidity meter (TES, TES-1361, Taipei, Taiwan), and air velocity was measured using a hot-wire anemometer (TSI-8386A, TSI Inc., Shoreview, MN, USA). The resulting measurements were summarized using the mean, standard deviation, and coefficient of variation. These tests were used to confirm that the chamber could provide sufficient environmental control for candle combustion.

### 2.3. Experimental Matrix and Operation

A paraffin-based scented candle containing 6% lavender essential oil was used for the PAH emission experiments as a representative commonly used formulation. Two environmental condition series were evaluated: RH was varied at 60%, 75%, and 90% under a fixed ACH of 0.5 h^−1^, while ACH was varied at 0.5, 1.0, and 2.0 h^−1^ under a fixed RH of 75%. Each condition was tested in triplicate. Three candles were burned simultaneously in each run to ensure sufficient PAH mass for sampling. Candle consumption rate (CCR) was determined from the difference in candle mass measured before and after each test.

Only normal burning conditions were included in this study. Normal burning was defined as a stable flame without visible soot or smoke. Sooting and smoldering were defined as unstable combustion accompanied by visible soot formation and white smoke emitted after flame extinction, respectively [18]. Before PAH sampling, air velocity, RH, CO_2_, and CO were continuously monitored using direct-reading instruments until the chamber approached steady-state operation. A 1.5 h sampling period was then initiated. Sampling was terminated if CO_2_ or CO varied by more than 10% during a test, to ensure consistent normal burning conditions.

CO_2_ and CO were measured using a portable flue gas analyzer (IMR 1100-2, IMR Environmental Equipment, St. Petersburg, FL, USA), with measurement ranges of 0–4000 ppm and 0–2000 ppm, respectively, and accuracies of ±5% and ±0.3%, respectively. Air velocity was measured using a hot-wire anemometer (TSI 8386A, TSI Inc., Shoreview, MN, USA), with a measurement range of 0.01–50.0 m/s and an accuracy of ±0.01%.

### 2.4. PAH Sampling

A PAH sampling train was installed in the sampling section of the chamber to characterize the PAH emission profiles and emission concentrations of the selected scented candles. Particle-bound and gas-phase PAHs were collected using a modified NIOSH Method 5515. The sampling train consisted of a filter cassette followed by a sorbent tube containing washed XAD-2 resin (3.5 g/0.5 g), operated at a flow rate of 2 L min^−1^.

Prior to sampling, all filters were cleaned with a solvent mixture of n-hexane and dichloromethane (1:1, *v*/*v*) for 24 h using a Soxhlet extractor. After sampling, particle-loaded filters were weighed using an electronic microbalance (MP 8-6, Sartorius AG, Göttingen, Germany) and then transported, together with the sorbent tubes, to the laboratory for PAH analysis. Particle-bound and gas-phase PAH concentrations were subsequently determined.

### 2.5. PAH Analysis

For PAH analyses, each sample collected was placed in a solvent solution (a mixture of *n*-hexane and dichloromethane; v:v = 500 mL:500 mL), and extracted in a Soxhlet extractor for 24 h. The extract was then concentrated, cleaned up, and re-concentrated to exactly 1.0 mL or 0.5 mL. PAH content was determined with a gas chromatograph (GC) (5890A, Hewlett-Packard Company, Palo Alto, CA, USA) equipped with a mass selective detector (MSD) (5972, Hewlett-Packard Company, Palo Alto, CA, USA) and a computer workstation. The GC/MS equipped with a Hewlett-Packard capillary column (HP Ultra 2—50 m × 0.32 mm × 0.17 μm) and an HP-7673A automatic sampler (7673A, Hewlett-Packard Company, Palo Alto, CA, USA) was operated under the following conditions: injection volume 1 μL, splitless injection at 310 °C, ion source temperature at 310 °C, oven from 50 °C to 100 °C at 20 °C/min; 100 °C to 290 °C at 3 °C/min; hold at 290 °C for 40 min. The masses of primary and secondary ions of PAHs were determined in the scan mode for pure PAH standards. Quantification of PAHs was performed in the same selected ion monitoring (SIM) mode.

The concentrations of 21 PAH species were determined, including naphthalene (Nap), acenaphthylene (AcPy), acenaphthene (Acp), fluorene (Flu), phenanthrene (PA), anthracene (Ant), fluoranthene (FL), pyrene (Pyr), cyclopenta(c,d)pyrene (CYC), benzo(a)anthracene (BaA), chrysene (CHR), benzo(b)fluoranthene (BbF), benzo(k)fluoranthene (BkF), benzo(e)pyrene (BeP), benzo(a)pyrene (BaP), perylene (PER), indeno(1,2,3,-cd)pyrene (IND), dibenzo(a,h)anthracene (DBA), benzo(b)chrycene (BbC), benzo(ghi)perylene (BghiP) and coronene (COR). Recovery efficiencies, determined by processing a solution containing known PAH concentrations following the same experimental procedure used for the treatment of samples, showed values from 0.760 to 1.071, with an average value of 0.854. The blank tests for PAHs were accomplished by using the same procedure as in the recovery-efficiency tests without adding the known standard solution before extraction. Field blanks, including glass-fiber filters and XAD-2 cartridges, showed no significant contamination (GC/MS integrated area < detection limit).

### 2.6. Data Analysis

Particle-bound and gas-phase total-PAHs were defined as the sums of the 21 target PAH species measured in the particle and gas phases, respectively. Total-PAHs were calculated as the sum of particle-bound and gas-phase PAHs. PAHs were further grouped by molecular weight into low-molecular-weight PAHs (LMW-PAHs; two- to three-ring PAHs), middle-molecular-weight PAHs (MMW-PAHs; four-ring PAHs), and high-molecular-weight PAHs (HMW-PAHs; five- to seven-ring PAHs). The concentrations of LMW-, MMW-, and HMW-PAHs were calculated as the sums of the corresponding PAH species within each group.

Emission factors (EFs) were calculated by normalizing PAH concentrations to the exhaust flow rate and candle consumption rate via Equation (1):

(1) $${EF}_{PAHi}=\frac{C_{i}\times Q}{CCR}$$

where

EF_PAH*i*_: the emission factor of PAH compound *i* (ng g^−1^);

C_i_: measured PAH concentrations of compound *i* (ng m^−3^);

Q: exhaust gas flow rate (m^3^ h^−1^);

CCR: the scented candle consumption rate (g h^−1^).

In principle, the carcinogenic potency of a given PAH compound can be assessed based on its benzo[*a*]pyrene equivalent concentration. Toxicity-weighted PAH concentrations were expressed as benzo[*a*]pyrene-equivalent concentrations (BaPeq). Calculation of the BaPeq concentration for a given PAH compound requires the use of the toxic equivalent factor (TEF), which represents the relative carcinogenic potency of the given PAH compound by reference to the specific compound BaP, to adjust its original concentration. Several proposals for TEFs [24,25,26] and compiled in our previous study [27] were adopted in the present study. On the basis of this TEF list, the carcinogenic potency of the total-PAHs (i.e., the total-BaPeq) can be assessed using Equation (2):

(2) $${Total-BaP}_{eq}=\sum_{i=1}^{21}C_{i}\times {TEF}_{i}$$

And the EF for the total-PAHs (EF_Total-PAHs_) and total-BaPeq (EF_Total-BaPeq_) can be assessed via Equations (3) and (4), respectively:

(3) $${EF}_{Total-PAHs}=\sum_{i=1}^{21}{EF}_{PAHi}$$

(4) $${EF}_{{Total-BaP}_{eq}}=\frac{{Total-BaP}_{eq}\times Q}{CCR}$$

For Statistical analysis, normality and homogeneity of variance were evaluated using the Shapiro–Wilk and Levene tests, respectively. Differences among air-exchange-rate conditions and among relative-humidity conditions were assessed by one-way analysis of variance (ANOVA) with Tukey’s honestly significant difference post hoc test; when the normality assumption was not met, the Kruskal–Wallis test with Dunn’s post hoc comparison was applied. A *p*-value < 0.05 was considered statistically significant. All analyses were performed in [SPSS v.26].

### 2.7. Conducting Health Risk Assessment for Scented Candle Users

In this study, aromatherapy workers were selected as the target population. Assuming daily scented candle use for 8 h, the incremental lifetime lung cancer risk (ILCR) associated with PAH exposure was assessed. The ILCR was estimated using a lifetime average daily dose (LADD) and cancer slope factor (CSF) approach, which can be expressed as Equation (5):

(5) ILCR=LADD×CSF

The LADD can be calculated via Equation (6):

(6) $$LADD=\frac{{C_{BaP}}_{eq}\times IR\times \frac{ET}{24}\times EF\times ED}{BW\times AT}$$

where

IR: inhalation rate (20 m^3^ day^−1^);

ET: daily exposure time (8 h day^−1^);

EF: exposure frequency (250 days year^−1^);

ED: exposure duration (40 years);

BW: body weight (70 kg);

AT: the averaging time for carcinogenic effects (days; 70 years = 25,550 days).

The C_BaPeq_ under each environmental condition was estimated from the measured EF_Total-BaPeq_ using a steady-state well-mixed room model as Equation (7):

(7) $$C_{{BaP}_{eq}}=\frac{G}{ACH\times V}$$

where the generation rate (G) is:

(8) $$G=EF_{{total-BaP}_{eq}}\times CCR\times CN$$

where

CCR: candle consumption rate (g hr^−1^);

CN: numbers of candles burning indoor.

A Monte Carlo simulation with 100,000 iterations was performed to propagate the uncertainty and variability of the input parameters into the exposure and risk estimates. Because aromatherapy is typically administered in small, enclosed treatment rooms, the room volume (V) was based on dimensions representative of such rooms. To reflect realistic variability in room size, V was treated as a uniform distribution spanning a single-occupancy treatment room (6 m × 4 m × 3 m; V = 72 m^3^) and a double-occupancy treatment room (6 m × 6 m × 3 m; V = 108 m^3^), and three of the selected scented candles (CN = 3) were assumed to burn simultaneously. The condition-specific inputs EF_total-BaPeq_ and CCR were each assigned normal distributions defined by the mean and standard deviation measured in the chamber experiments. To ensure a health-protective estimate, the BaPeq concentrations were computed using the most conservative (highest) TEFs reported in the published literature. The ILCR was estimated separately for each ACH and RH condition.

## 3. Results

### 3.1. Stability and Uniformity of Exposure Chamber

**Figure 2:**
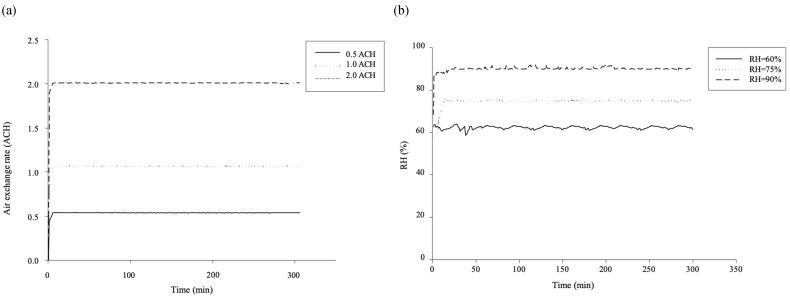
Temporal stability of (**a**) air exchange rate (ACH) and (**b**) relative humidity (RH) in the exposure chamber.

The exposure chamber demonstrated satisfactory stability and spatial uniformity under the tested environmental conditions. The stability of air exchange rate (ACH) and relative humidity (RH) is presented in Figure 2a and Figure 2b, respectively. For the three tested ACH levels, the system reached steady-state conditions within approximately 10 min and remained stable throughout the duration of the experiments. Similarly, for RH control, the chamber achieved steady state within 10 min at RH = 90% and within 20 min at RH = 75%. However, at RH = 60%, a longer equilibration time of approximately 50 min was required to reach steady state, and slightly greater fluctuations were observed. Nevertheless, all environmental parameters remained well-controlled and exhibited acceptable stability over extended experimental periods.

The spatial uniformity of environmental conditions within the chamber was evaluated by measuring RH and ACH at three different sampling layers and locations on each layer (Table 1). For RH, the measured values across the three layers were highly consistent. At target RH levels of 60%, 75%, and 90%, the observed means ranged from 60.6 to 61.4%, from 74.1 to 76.0%, and from 89.4 to 90.9%, respectively (Table 1). The differences among sampling layers were within ±2% RH, and the corresponding standard deviations within each layer were within 3.64–6.82%, indicating minimal spatial variability and good humidity uniformity throughout the chamber.

Similarly, ACH measurements showed strong agreement across the three sampling layers. At target ACH values of 0.5, 1.0, and 2.0 h^−1^, the measured means ranged from 0.53 to 0.56 h^−1^, from 1.02 to 1.13 h^−1^, and from 2.08 to 2.16 h^−1^, respectively. The relative variation among locations was within approximately ±5%, and the corresponding standard deviations within each layer were low (≤0.04 h^−1^), demonstrating stable and spatially uniform ventilation conditions in the chamber.

Overall, both RH and ACH exhibited high spatial consistency with low variability across measurement locations. These results confirm that the environmental control reproducibility was sufficient to support reliable inter-condition comparisons of PAH emissions.

### 3.2. PAH Emissions with Different Air Exchange Rate

The influence of air exchange rate (ACH) on PAH emissions was evaluated under three ventilation conditions (ACH = 0.5, 1.0, and 2.0 h^−1^) at a constant relative humidity of 75%. As shown in Table 2, total PAH concentrations exhibited a clear dependence on ACH, with mean values of 2799, 1112, and 1218 ng m^−3^ at 0.5 ACH, 1.0 ACH, and 2.0 ACH, respectively. Statistical analysis indicated that the total PAH concentration at 0.5 ACH was significantly higher than those at 1.0 and 2.0 ACH (*p* < 0.001), whereas no significant difference was observed between 1.0 and 2.0 ACH (*p* = 0.83).

To eliminate the dilution effect caused by ventilation, PAH emissions were further normalized to the mass of fuel consumed and expressed as EFs (ng g^−1^). The corresponding EF_total PAHs_ were 64.6, 19.9, and 21.9 ng g^−1^ at 0.5, 1.0, and 2.0 ACH, respectively. Both total PAH concentrations and EFs followed the same trend: 0.5 ACH > 2.0 ACH > 1.0 ACH.

Considering the toxicity-weighted concentrations, the Total-BaPeq were 22.1, 10.9, and 8.41 ng m^−3^ at 0.5, 1.0, and 2.0 ACH, respectively, while the corresponding EF_Total-BaPeq_ values were 0.511, 0.194, and 0.151 ng g^−1^. Both Total-BaPeq and EF_Total-BaPeq_ decreased with increasing ACH, following the trend: 0.5 ACH > 1.0 ACH > 2.0 ACH.

However, based on the post hoc comparison, the 0.5 ACH condition showed significantly higher values than the 1.0 and 2.0 ACH conditions (*p* < 0.001), while the difference between 1.0 and 2.0 ACH was not statistically significant for both EF_total-PAHs_ and EF_total-BaPeq_ (*p* = 0.86 and 0.54, respectively).

At the compound level, LMW PAHs, particularly naphthalene (NaP), dominated total PAH emissions across all ACH conditions. LMW PAHs accounted for approximately 87.0%, 87.1%, and 90.5% of total PAHs at 0.5, 1.0, and 2.0 ACH, respectively. MMW PAHs contributed 10.6%, 7.78%, and 7.26%, while HMW PAHs constituted only a minor fraction (2.43%, 5.22%, and 2.29%, respectively). Overall, the compositional distribution of PAHs remained relatively consistent across ACH conditions, although the relative contribution of HMW PAHs varied slightly and remained minor.

Regarding gas–particle partitioning, LMW PAHs were the dominant homologues in the gas phase, accounting for 80.2%, 81.8%, and 82.3% at 0.5, 1.0, and 2.0 ACH, respectively. In contrast, MMW and HMW PAHs exhibited relatively lower gas-phase fractions across all tested ACH conditions. The gas-phase fractions of HMW PAHs were 38.8%, 57.8%, and 28.6% at 0.5, 1.0, and 2.0 ACH, respectively, suggesting greater variability in gas–particle partitioning for heavier PAHs. These observations might be attributable to the lower volatility associated with higher molecular weight compounds.

### 3.3. PAH Emissions with Different Relative Humidity

The effect of relative humidity (RH) on PAH emissions was evaluated under three humidity conditions (RH = 60%, 75%, and 90%) at a constant ACH of 0.5 h^−1^. As shown in Table 3, total PAH concentrations varied with RH, with mean values of 1045, 2799, and 1303 ng m^−3^ at RH levels of 60%, 75%, and 90%, respectively. The highest PAH concentration was observed at RH = 75%, while lower concentrations were measured at both lower and higher humidity conditions. Statistical analysis indicated that RH = 75% had significantly higher total PAH concentrations than RH = 60% and RH = 90% (*p* < 0.001), whereas no significant difference was observed between RH = 60% and RH = 90% (*p* = 0.35).

To account for the effect of fuel consumption, the corresponding EF_Total-PAHs_ were 18.0, 64.6, and 30.8 ng g^−1^ at RH of 60%, 75%, and 90%, respectively, following the same trend as the concentration data (75% > 90% > 60%). The post hoc comparison showed that RH = 75% was significantly higher than RH = 60% and RH = 90% (*p* < 0.001), and that RH = 90% was significantly higher than RH = 60% (*p* = 0.042). These results indicate that PAH formation was enhanced under intermediate humidity conditions, while both lower and higher RH levels resulted in reduced emissions.

For the toxicity-weighted concentrations, total-BaPeq concentrations were 7.56, 22.1, and 6.95 ng m^−3^ at RH of 60%, 75%, and 90%, respectively, while EF_Total-BaPeq_ values were 0.130, 0.511, and 0.164 ng g^−1^. Post hoc comparisons showed that RH = 75% was significantly higher than RH = 60% and RH = 90% for both Total-BaPeq and EF_Total-BaPeq_ (*p* < 0.001), while no significant difference was observed between RH = 60% and RH = 90% for Total-BaPeq (*p* = 0.94) or EF_Total-BaPeq_ (*p* = 0.66). These results suggest that intermediate humidity substantially enhanced toxicity-weighted PAH emissions, whereas the difference between low and high RH conditions was not statistically significant.

At the compound level, LMW PAHs remained dominant across all RH conditions, accounting for 88.7%, 87.0%, and 92.3% of total PAHs at RH = 60%, 75%, and 90%, respectively. MMW PAHs contributed 8.0%, 10.6%, and 6.7%, while HMW PAHs represented relatively small fractions of 3.2%, 2.4%, and 1.0%, respectively. Compared to RH = 75%, both MMW and HMW PAHs decreased at RH = 90%, suggesting reduced higher molecular weight PAH formation under high humidity.

Regarding gas–particle partitioning, the gas-phase fraction of total LMW PAH emissions remained relatively stable across RH conditions, accounting for 80.9%, 80.6%, and 83.6% at RH levels of 60%, 75%, and 90%, respectively. Consistent with the previous results, both MMW and HMW PAHs exhibited relatively lower gas-phase fractions under all tested RH conditions, with HMW-PAHs showing the lowest gas-phase fraction. These results are primarily attributable to the lower volatility associated with compounds of higher molecular weight.

### 3.4. Combustion Characteristics Under Different Environmental Conditions

The combustion characteristics of the candles under different ventilation and humidity conditions are summarized in Table 4. Variations in oxygen consumption, gaseous emission factors (CO and CO_2_), flame temperature, and candle combustion rate were observed across the tested conditions, indicating that both ACH and RH significantly influenced combustion behavior.

At a constant ACH of 0.5 h^−1^, increasing RH from 60% to 75% resulted in a marked decrease in oxygen consumption rate from 2.0 × 10^−3^ to 0.7 × 10^−3^ m^3^ g^−1^, accompanied by an increase in CO emission factor from 1.7 × 10^−6^ to 4.0 × 10^−6^ m^3^ g^−1^ and CO_2_ emission factor from 4.3 × 10^−4^ to 1.29 × 10^−3^ m^3^ g^−1^. Simultaneously, the flame core temperature decreased from 626 to 582 °C, and the combustion rate declined from 1.74 to 1.29 g h^−1^. These results suggest that increasing RH at an intermediate humidity (RH = 75%), combustion efficiency decreased, likely due to the cooling effect of water vapor and altered combustion kinetics. However, at higher humidity (RH = 90%), the oxygen consumption rate increased to 1.5 × 10^3^ m^3^ g^−1^ compared to RH = 75%, while CO and CO_2_ emission factors remained elevated (4.2 × 10^−6^ and 1.0 × 10^−3^ m^3^ g^−1^, respectively). The flame temperature further decreased to 567 °C, and the combustion rate remained relatively low (1.26 g h^−1^). This indicates that high humidity continued to suppress flame temperature.

Under increasing ACH at a constant RH of 75%, a different trend was observed. The combustion rate increased substantially from 1.29, 3.35, and 6.57 g h^−1^ at 1.0, 0.5, and 2.0 ACH, respectively, indicating enhanced fuel consumption with increased air supply. Correspondingly, flame temperature reached its maximum at 1.0 ACH (633 °C) and slightly decreased at 2.0 ACH (612 °C), while excessive airflow may induce flame disturbance.

In terms of gaseous emissions, CO emission factors decreased with increasing ACH (from 4.0 × 10^−6^ to 2.7 × 10^−6^ m^3^ g^−1^), while CO_2_ emission factors slightly decreased from 1.29 × 10^−3^ to 8.4 × 10^−4^ m^3^ g^−1^. The reduction in CO emissions with increasing ACH indicates improved combustion completeness due to enhanced oxygen availability. Meanwhile, the relatively stable oxygen consumption rates (1.2–1.3 × 10^−3^ m^3^ g^−1^) suggest that combustion efficiency reached a plateau at higher ventilation levels.

### 3.5. Predicted PAH Exposure Concentrations and Incremental Cancer Risk

The BaPeq exposure concentrations and the corresponding incremental lifetime lung cancer risk (ILCR) under different environmental conditions are summarized in Table 5. The mean BaPeq exposure concentrations ranged from 0.015 to 0.046 ng m^−3^ across all tested conditions. Among them, the highest exposure level was observed at 0.5 ACH and RH = 75% (0.046 ± 0.017 ng m^−3^), whereas substantially lower concentrations were found at both lower and higher humidity conditions (0.015 ng m^−3^ at RH = 60% and 90%). Under increased ventilation conditions at RH = 75%, BaPeq concentrations decreased to 0.014 and 0.011 ng m^−3^ at 1.0 and 2.0 ACH, respectively, indicating a clear mitigating effect of ventilation on carcinogenic PAH exposure.

The corresponding ILCR values ranged from 2.19 × 10^−7^ to 7.66 × 10^−7^ for mean exposure, with 95th percentile values ranging from 3.36 × 10^−7^ to 1.20 × 10^−6^. The highest cancer risk was observed at 0.5 ACH and RH = 75%, consistent with the condition exhibiting the highest BaPeq concentration. The 95th percentile ILCR under this condition (1.20 × 10^−6^) slightly exceeded the commonly accepted risk threshold of 1 × 10^−6^.

## 4. Discussion

### 4.1. Comparison with Previous Studies

To the best of our knowledge, this study is the first to systematically investigate PAH emission characteristics from candle combustion under controlled variations in both air exchange rate (ACH) and relative humidity (RH). Previous studies on candle emissions have primarily focused on PAH concentrations under fixed indoor conditions without explicitly addressing the influence of environmental parameters such as ventilation or humidity [9,12,28]. While the effects of RH on PAH emissions have been examined for other combustion sources such as incense [23], a combined evaluation of ACH and RH for candle-derived PAHs has not been reported.

In the present study, the validity of the experimental environment system was confirmed through comprehensive chamber stability and uniformity tests. Both temporal stability and spatial uniformity of ACH and RH demonstrated consistent control throughout the experiments, which is comparable to or faster than typical environmental chamber studies for indoor emission experiments [12,29]. Although slightly larger temporal fluctuations were observed under low humidity conditions (RH = 60%), this was primarily attributed to the inlet air humidity being close to the target RH, causing intermittent operation of the humidification system. Overall, these results validate the use of chamber-derived emission factors and support reliable inter-condition comparisons of PAH emissions.

The total PAH and BaPeq EFs obtained in this study ranged from 17.6 to 72.9 ng g^−1^ and from 0.097 to 0.596 ng g^−1^, respectively, which are generally comparable to values reported in previous studies. For example, Derudi et al. [28] reported total PAH EFs ranging from 26.6 to 152 ng g^−1^ for container candles made of different paraffin waxes, tested in a small laboratory test chamber at an ACH of approximately 4 h^−1^. The higher ventilation rate and the use of multiple paraffin formulations likely contribute to the broader EF range relative to the single formulation tested in the present study. The PAH profiles reported in the study were also dominated by gaseous, low-molecular-weight species, consistent with the compositional characteristics observed in the present study.

Salthammer et al. [13] investigated PAH emissions from both scented and unscented candles in a chamber operated at 2 ACH, reporting emission rates of 172–1286 ng h^−1^ and 118 ng h^−1^ per candle, respectively. When converted to emission factors, the corresponding EF for the selected scented candles is estimated to be approximately 37–359 ng g^−1^, which is slightly higher than the values obtained in this study. Because that study sampled a wide range of wax and fragrance combinations, this difference might be attributed to variations in fragrance composition and additive content, rather than a true difference in emission strength.

Orecchio [9] reported total PAH EFs in the range of 2.3–49.8 ng g^−1^ for twelve types of candles in market, which are comparable to the lower range of the present study; the wider span reflects the formulation variability inherent in surveying many commercial products. In addition, Andersen et al. [11] evaluated candle emissions under conditions with airflow disturbance in a 2 ACH chamber, and reported total PAH and BaPeq emission rate, which could convert to EFs of 69–192 ng g^−1^ and 0.125–0.7 ng g^−1^, respectively. These higher values are consistent with enhanced flame instability and incomplete combustion under disturbed airflow, in contrast to the controlled, well-mixed conditions and normal burning regime used here, and underscore the strong dependence of emissions on local airflow and combustion stability. The magnitude of the PAH emission factors reported in this study falls within the range of previous literature, but the wide spread of reported values is largely attributable to differences in candle formulation (wax, wick, and fragrance), chamber volume and mixing, ventilation rate, sampling methodology, and combustion stability. Cross-study comparisons of absolute emission factors should therefore be interpreted with caution. Crucially, none of the existing studies have systematically examined the effects of ventilation and humidity on candle PAH emissions.

### 4.2. Possible Mechanistic Interpretation of the ACH Effects

For the influence of ACH, the results suggest that ACH may influence PAH emissions through both dilution and combustion-related effects. Although a higher ACH is expected to lower indoor pollutant concentrations through enhanced dilution, the variation in emission factors suggests that ACH may also affect the combustion process itself. At the lowest ACH (0.5 h^−1^), a restricted oxygen supply may have promoted incomplete combustion and thus higher PAH emissions; this interpretation is consistent with the comparatively higher CO emission factor and lower O_2_-consumption efficiency observed under this condition (Table 4). At a moderate ACH (1.0 h^−1^), the total PAH and BaPeq emission factors decreased substantially to 19.9 and 0.194 ng g^−1^, respectively, which is consistent with more complete combustion under greater oxygen availability. Such behavior would be in line with combustion theory, in which a sufficient oxygen supply promotes oxidation and suppresses the formation of intermediate products such as PAHs [14]. At the highest ACH (2.0 h^−1^), the EF_Total-PAHs_ increased slightly (21.9 ng g^−1^) relative to 1.0 h^−1^, which might be associated with airflow-induced disturbances that affect flame stability and could produce localized incomplete combustion [11]. In contrast, the EF_Total-BaPeq_ decreased further to 0.151 ng g^−1^ at 2.0 h^−1^. This pattern suggests that, although increased airflow may slightly raise total PAH emissions, the additional PAHs were predominantly LMW species of relatively low toxicity, whereas the formation of HMW PAHs, which dominate total toxicity, appears to be suppressed under higher ventilation. This is consistent with previous reports that improved combustion conditions tend to inhibit the formation of high-ring PAHs [11].

Because LMW PAHs are generally associated with lower combustion temperatures and shorter residence times [14], the combustion parameters in Table 4 provide further, if indirect, support for this interpretation. At 1.0 h^−1^, the flame-core temperature reached its highest value (633 ± 18 °C) and coincided with the lowest PAH and BaPeq emission factors; although the CO emission factor remained moderate (3.8 × 10^−6^ m^3^ g^−1^) and oxygen consumption was intermediate (4.3 × 10^−4^ m^3^ g^−1^), the elevated flame temperature is consistent with conditions that favour the oxidation of PAH precursors. At 2.0 h^−1^, oxygen consumption increased (1.3 × 10^−3^ m^3^ g^−1^) and the CO emission factor decreased (2.7 × 10^−6^ m^3^ g^−1^), which may reflect more efficient oxidation; the candle consumption rate nearly doubled (6.57 ± 0.67 g h^−1^), whereas the flame-core temperature decreased to 612 ± 9 °C, possibly because of the higher airflow.

### 4.3. Possible Mechanistic Interpretation of the RH Effects

The effect of RH on PAH emissions was non-monotonic, with the highest emissions at RH = 75% and lower emissions at both RH = 60% and RH = 90%. This behavior can be rationalized by two well-documented features of PAH and soot formation. First, PAH formation is intrinsically non-monotonic in temperature: both PAH and soot yields are widely reported to follow a “bell-shaped” dependence on temperature [30,31]. Second, water vapor modulates the flame both thermally and chemically. Water vapor acts as a heat sink that lowers the flame temperature, and it also exerts a chemical effect of comparable magnitude through the reaction OH + H_2_ ↔ H + H_2_O, whereby the resulting reduction in H-radical concentration lowers PAH concentrations and soot inception rates [32]. Because increasing RH progressively lowers the flame temperature, varying humidity effectively moves the system along the bell-shaped temperature–yield curve, while the chemical suppression by water vapor becomes increasingly important only as humidity rises towards the higher end of the range.

These two effects together might account for the observed pattern. At low humidity (RH = 60%), the higher flame temperature (626 °C) places the system on the high-temperature, oxidation-dominated side of the curve, consistent with the lowest EF_total-PAHs_ and EF_Total-BaPeq_ and with more complete combustion, the CO emission factor was lowest (1.7 × 10^−6^ m^3^ g^−1^), the CO_2_ emission factor comparatively higher (1.2 × 10^−3^ m^3^ g^−1^), and the combustion rate slightly higher, in line with the expectation that sufficiently high temperatures favor the oxidation of aromatic intermediates rather than their accumulation [14]. At intermediate humidity (RH = 75%), the flame temperature remains high enough to sustain pyrolytic PAH formation while the chemical suppression by water vapor is not yet dominant, placing the system near the top of the bell-shaped curve and favoring the net accumulation of PAHs, which is reflected in the peak emissions. At high humidity (RH = 90%), both the thermal-quenching and the H-radical-scavenging effects intensify, lowering the flame temperature to 567 °C and suppressing PAH formation; consistent with this lower-temperature regime, a higher fraction of LMW PAHs was observed than at RH = 60%. A broadly similar tendency has been reported for incense combustion [23], although differences in fuel composition and burning behavior between incense and candles warrant caution in any direct comparison. This interpretation is consistent with the measured flame-temperature and CO/CO_2_ trends.

However, it should be noted that combustion chemistry was not directly characterized in this study. Therefore, the mechanisms proposed in Section 4.2 and Section 4.3 are based on indirect combustion indicators and should be interpreted as plausible hypotheses rather than demonstrated mechanisms. Further confirmation is required by using direct combustion diagnostics such as combustion efficiency, flame structure, soot yield, and carbon balance.

### 4.4. Implications for Exposure and Public Health

From a cancer risk perspective, ventilation exhibited a clear mitigating effect on cancer risk. Although higher EF were observed at ACH = 1 and 2 under RH = 75% compared to ACH = 0.5 under RH = 60% and 90%, increased ventilation effectively diluted BaPeq concentrations, resulting in lower exposure levels and reduced ILCR. Overall, most of the estimated ILCR values remained below the commonly accepted risk threshold of 1 × 10^−6^, indicating that the cancer risk associated with candle use under the tested conditions is generally within acceptable limits. However, the results also demonstrate that under conditions of low ventilation and intermediate humidity, the risk may exceed this threshold.

Among all conditions, the highest cancer risk occurred at 0.5 ACH and RH = 75%, where the 95th-percentile ILCR (1.20 × 10^−6^) exceeded the commonly accepted threshold of 1 × 10^−6^. Although this exceedance is modest and reflects a conservative occupational scenario, it indicates that long-term, intensive use of scented candles in poorly ventilated, intermediate-humidity environments might pose a carcinogenic risk that should not be dismissed. In addition, this estimate accounts only for PAHs; scented candle combustion also emits other carcinogenic or irritant species, such as benzene and formaldehyde [13,33,34], which would add to the total incremental cancer risk.

These findings highlight the importance of adequate ventilation in reducing exposure to carcinogenic PAHs during candle burning. They also emphasize the need to consider both ventilation and humidity when evaluating indoor emission sources, as these factors may influence emission characteristics beyond simple dilution effects.

### 4.5. Limitations

Several limitations should be acknowledged. First, only a single type of candle (paraffin-based candle with 6% Lavender essential oil) was tested, and emissions may vary with different wax compositions, wick characteristics, fragrance loading, or additives. Extrapolation of the present results to other candle types should be made with caution, and confirmation across a wider range of formulations is warranted in future work. Second, the experiments were conducted in a relatively small chamber with three candles burning simultaneously, under a controlled and well-mixed airflow field. While this design was deliberately chosen to isolate the effects of ACH and RH under reproducible conditions, it may not fully capture the airflow patterns of larger indoor environments, where drafts, thermal convection, and HVAC supply could alter the local flow around the flame, thereby affecting flame stability and local oxygen availability. Consequently, while the qualitative trends and underlying combustion mechanisms are expected to be broadly transferable, the absolute emission factors and the precise shape of the ACH response may be influenced by the chamber configuration and should be extrapolated to larger or differently ventilated rooms with appropriate caution. Further field measurements in occupied rooms would help to confirm and refine these relationships. Third, the experiments were conducted under steady combustion conditions. In contrast, actual candle use may involve transient behaviors such as the final stage of burning when the candle is nearly consumed, which could influence emission profiles. Finally, this study quantified PAHs only, whereas scented candles co-emit particulate matter, ultrafine particles, and carcinogenic VOCs such as benzene and formaldehyde. The PAH-based risk estimated here therefore captures only part of the total exposure, and a full evaluation of the incremental cancer risk from scented candle use should integrate these co-emitted pollutants under controlled ventilation and humidity conditions in future work.

## 5. Conclusions

This study established a stable exposure chamber and demonstrated that PAH emissions from the selected scented candle combustion are strongly affected by indoor environmental conditions. The chamber provided stable and spatially uniform control of ACH and RH, enabling reliable comparisons across test conditions. PAH emissions were generally highest under low ventilation and intermediate humidity. At RH = 75%, total PAH concentrations and EFs followed the order 0.5 ACH > 2.0 ACH > 1.0 ACH, whereas total- and EF total- BaPeq values decreased with increasing ACH (i.e., 0.5 ACH > 1.0 ACH > 2.0 ACH).

At ACH = 0.5 h^−1^, PAH emissions showed a nonlinear response to RH, with the highest EF of total-PAHs and total-BaP_eq_ observed at RH = 75%, followed by 90% and 60%.

PAH profiles were dominated by gaseous low-molecular-weight compounds, but toxicity-weighted risk was controlled by the formation and partitioning of more toxic higher-ring species. Screening-level ILCR estimates were generally below 10^−6^; however, the 95th percentile estimate under 0.5 ACH and RH = 75% reached 1.20 × 10^−6^. These results highlight the importance of adequate ventilation during scented candle use and show that humidity should be considered when evaluating indoor combustion emissions. Product testing and consumer guidance should account for realistic environmental conditions rather than relying only on formulation-based emission estimates.

## Figures and Tables

**Table 1:** Spatial uniformity of air exchange rate (ACH) and relative humidity (RH) across different sampling layers within the exposure chamber.

Environment Factor		Layers (Mean ± SD)		
		1st	2nd	3rd
RH (%)	60	61.4 ± 5.48	61.3 ± 3.77	60.6 ± 4.14
	75	75.3 ± 6.82	74.1 ± 5.29	76.0 ± 6.61
	90	90.9 ± 3.64	90.4 ± 4.83	89.4 ± 6.70
ACH (h^−1^)	0.5	0.55 ± 0.03	0.53 ± 0.04	0.56 ± 0.02
	1	1.13 ± 0.03	1.02 ± 0.02	1.07 ± 0.02
	2	2.11 ± 0.04	2.16 ± 0.02	2.08 ± 0.03

**Table 2:** PAH emission characteristics under different air exchange rate (ACH) conditions at constant relative humidity (RH = 75%).

Compounds	ACH = 0.5 h^−1^				ACH = 1.0 h^−1^				ACH = 2.0 h^−1^			
	Range	Mean	RSD (%)	Gas Fraction (%)	Range	Mean	RSD (%)	Gas Fraction (%)	Range	Mean	RSD (%)	Gas Fraction (%)
NaP	1714–2966	2102	9.70	84.2	666–886	745	16.4	89.3	706–1042	913	19.8	88.4
AcPY	46.9–61.4	52.9	14.3	57.0	33.2–35.5	34.7	3.80	59.1	23.5–26.5	24.6	6.57	51.6
Acp	108–133	119	11.1	53.9	52.3–69.4	62.0	14.1	49.0	36.8–34.5	35.7	3.26	50.7
Flu	107–150	131	17.1	55.0	24.6–36.9	31.1	19.9	60.8	31.9–37.7	34.9	8.25	55.6
PA	108–133	117	11.1	55.7	57.8–59.6	58.8	1.50	60.2	44.6–59.4	53.4	14.6	51.9
Ant	64.1–174	133	45.1	63.1	33.1–40.5	36.1	10.9	60.4	48.7–32.5	40.6	20.0	54.9
FL	117–147	128	12.9	56.8	27.3–34.6	31.0	11.8	61.9	25.1–31.9	28.7	11.8	51.9
Pyr	97.8–108	103	5.10	45.1	22.9–23.6	23.1	1.90	50.6	21.3–33.7	28.2	22.5	52.5
CYC	6.55–35.9	22.7	65.6	28.5	16.1–37.4	29.9	40.1	62.2	0.24–15.8	10.5	84.6	0.00
BaA	32.0–46.9	37.8	21.1	49.7	9.10–20.2	14.8	37.5	12.5	ND–15.9	10.6	85.8	0.00
CHR	25.7–30.0	27.7	7.80	53.0	10.1–23.9	17.7	39.8	36.5	19.7–21.7	20.9	4.97	48.3
BbF	34.4–46.3	38.4	17.7	51.7	16.5–38.3	27.8	39.4	54.0	3.82–22.1	15.7	65.6	27.1
BkF	ND–12.7	6.88	92.9	0.00	ND	–	–	–	0.72–1.97	1.51	45.3	42.1
BeP	ND	–	–	–	ND–0.29	0.18	79.9	0.00	ND	–	–	–
BaP	ND	–	–	–	0.18–0.25	0.21	16.6	0.00	ND	–	–	–
PER	ND	–	–	–	ND	–	–	–	ND	–	–	–
IND	ND	–	–	–	ND	–	–	–	ND	–	–	–
DBA	ND	–	–	–	ND	–	–	–	ND	–	–	–
Bbc	ND	–	–	–	ND	–	–	–	ND	–	–	–
BghiP	ND	–	–	–	ND	–	–	–	ND	–	–	–
COR	ND	–	–	–	ND	–	–	–	ND	–	–	–
DBP	ND	–	–	–	ND	–	–	–	ND	–	–	–
LMW	2153–2754	2434	12.4	80.2	895–1121	968	13.6	81.8	888–1233	1102	17.0	82.3
MMW	284–313	297	5.04	51.5	73.0–97.3	86.6	14.3	45.4	73.0–100	88.4	15.9	45.0
HMW	41.0–90.2	68.1	36.7	38.8	45.1–76.3	58.1	27.9	57.8	6.10–23.6	17.1	67.7	28.6
Total-PAHs	2537–3140	2799	11.0	75.1	1006–1294	1112	14.2	77.8	1027–1344	1218	13.8	78.2
Total-BaP_eq_	15.8–25.8	22.1	13.6	53.3	6.96–13.9	10.9	18.8	46.6	5.39–10.1	8.41	17.0	36.9
EF_Total-PAHs_	58.9–72.9	64.6	11.0	74.9	18.0–23.1	19.9	14.2	77.4	18.5–24.2	21.9	13.8	78.1
EF_Total-BaPeq_	0.37–0.60	0.51	13.6	53.8	0.13–0.25	0.19	18.8	46.8	0.10–0.18	0.15	17.0	36.8

**Table 3:** PAH emission characteristics under different relative humidity (RH) conditions at a constant air exchange rate (ACH = 0.5 h^−1^).

Compounds	RH = 60%				RH = 75%				RH = 90%			
	Range	Mean	RSD (%)	Gas Fraction (%)	Range	Mean	RSD (%)	Gas Fraction (%)	Range	Mean	RSD (%)	Gas Fraction (%)
NaP	686–807	764	8.91	86.0	1714–2966	2102	9.70	84.2	953–1143	1048	12.8	88.1
AcPY	14.5–18.6	16.3	12.8	64.4	46.9–61.4	52.94	14.3	57.0	11.6–15.1	13.4	18.4	53.9
Acp	34.0–35.0	34.5	1.48	60.9	108–133	119.4	11.1	53.9	21.3–29.5	25.3	22.7	54.2
Flu	25.3–36.1	30.3	17.9	55.4	107–150	131.96	17.1	55.0	23.9–35.5	28.7	23.6	53.3
PA	22.2–57.9	45.6	44.6	57.5	108–133	117.86	11.1	55.7	49.7–55.8	52.7	8.20	53.7
Ant	34.3–38.9	36.3	6.58	51.0	64.1–174	133.13	45.1	63.1	28.8–38.5	33.6	20.5	54.5
FL	27.2–35.4	31.5	13.0	55.6	117–147	128.35	12.9	56.8	23.1–25.3	24.2	6.60	57.0
Pyr	27.6–37.1	31.0	17.1	50.3	97.8–108	103.29	5.10	45.1	22.2–29.9	26.1	20.8	54.8
CYC	3.20–15.2	10.4	61.1	20.2	6.55–35.9	22.74	65.6	28.5	21.2–23.3	22.3	6.60	61.0
BaA	3.30–15.7	10.7	61.1	20.2	32.0–46.9	37.83	21.1	49.7	ND	–	–	–
CHR	10.6–11.0	10.8	1.94	20.0	25.7–30.0	27.73	7.80	53.0	10.9–14.3	12.6	19.0	44.2
BbF	16.6–17.3	17.0	1.96	19.9	34.4–46.3	38.46	17.7	51.7	ND–17.1	–	–	–
BkF	ND–15.5	6.20	132	32.7	ND–12.7	6.88	92.9	0.00	0.19–14.3	5.03	–	0.04
BeP	ND–0.01	0.01	173	0.00	ND	–	–		ND	–	–	–
BaP	ND	–	–		ND	–	–		ND	–	–	–
PER	ND	–	–		ND	–	–		ND	–	–	–
IND	ND	–	–		ND	–	–		ND	–	–	–
DBA	ND	–	–		ND	–	–		ND	–	–	–
Bbc	ND	–	–		ND	–	–		ND	–	–	–
BghiP	ND	–	–		ND	–	–		ND	–	–	–
COR	ND	–	–		ND	–	–		ND	–	–	–
DBP	ND	–	–		ND	–	–		ND	–	–	–
LMW	872–969	927	5.41	80.9	2153–2754	2434	12.4	80.6	801–1316	1202	17.7	83.6
MMW	69.6–96.7	84.0	16.2	44.5	284–313	297	5.04	51.5	73.0–90.4	87.4	13.3	53.9
HMW	23.3–45.6	33.6	33.5	22.4	41.0–90.2	68.1	36.7	38.8	7.88–23.6	12.4	66.9	2.5
Total-PAHs	1014–1086	1045	3.59	76.1	2537–3140	2799	11.0	75.1	1168–1438	1303	14.6	80.9
Total-BaP_eq_	5.56–9.56	7.56	25.8	35.5	15.8–25.8	22.1	13.6	53.3	5.79–10.4	6.95	13.2	40.2
EF_Total-PAHs_	17.6–18.9	18.0	3.59	76.6	58.9–72.9	64.6	11.0	74.9	27.6–34.0	30.8	14.6	80.8
EF_Total-BaPeq_	0.10–0.17	0.13	25.8	35.3	0.37–0.60	0.51	13.6	53.8	0.14–0.25	0.16	13.2	40.7

**Table 4:** Characteristics of the selected scented candle combustions under different air exchange rates (ACH) and relative humidity (RH) conditions.

Burning Conditions	O_2_ Consumption rate (×10^−3^, m^3^ g^−1^)	CO EF (×10^−6^, m^3^ g^−1^)	CO_2_ EF (×10^−4^, m^3^ g^−1^)	Flame Core Temperature (°C)	Candle Combustion Rate (g h^−1^ Per Candle)
0.5 ACH RH = 60%	2.0 ± 0.22	1.7 ± 0.14	12 ± 1.44	626 ± 8	1.74 ± 0.09
0.5 ACH RH = 75%	0.7 ± 0.09	4.0 ± 0.39	4.3 ± 0.51	582 ± 8	1.29 ± 0.07
0.5 ACH RH = 90%	1.5 ± 0.03	4.2 ± 0.39	10 ± 0.16	567 ± 47	1.26 ± 0.1
1.0 ACH RH = 75%	1.2 ± 0.08	3.8 ± 0.23	7.8 ± 0.55	633 ± 18	3.35± 0.22
2.0 ACH RH = 75%	1.3 ± 0.08	2.7 ± 4.5	8.2 ± 0.43	612 ± 9	6.57± 0.67

**Table 5:** The BaPeq exposure concentrations (C_BaPeq_) and the corresponding incremental lifetime cancer risk (ILCR) under different environmental conditions.

Environment Conditions	CBaPeq (ng m^−3^)		ILCR	
	Mean	SD	Mean	95th%–Tile
0.5 ACH RH 60%	0.015	0.007	2.55 × 10^−7^	4.80 × 10^−7^
0.5 ACH RH 75%	0.046	0.017	7.66 × 10^−7^	1.20 × 10^−6^
0.5 ACH RH 90%	0.015	0.006	2.46 × 10^−7^	4.04 × 10^−7^
1.0 ACH RH 75%	0.014	0.009	2.31 × 10^−7^	3.98 × 10^−7^
2.0 ACH RH 75%	0.011	0.007	2.19 × 10^−7^	3.36 × 10^−7^

## Data Availability

The raw data supporting the conclusions of this article will be made available by the authors on request.
